# The New York Institute of Stomatology

**Published:** 1904-01

**Authors:** 

**Affiliations:** The New York Institute of Stomatology


### Reports of Society Meetings.

### THE NEW YORK INSTITUTE OF STOMATOLOGY.

A REGULAR meeting of the Institute was held at the "Chelsea," No. 222 West Twenty-third Street, New York, on Tuesday, October 6, 1903, the President, Dr. J. Morgan Howe, in the chair.

The minutes of the last meeting were read and approved.

The President stated that since the last meeting of the Institute one of the fellow-members, Dr. Louis Shaw, had passed away. A committee was appointed to draft suitable resolutions of regret and respect.

### COMMUNICATIONS ON THEORY AND PRACTICE.

Dr. Stockton presented a case of irregularity upon which he desired suggestions. Dr. Stockton stated that the question had arisen in his mind whether it would not be better, considering the fact that the boy was in school and objected very much to wearing an appliance, to extract a single tooth and complete the reduction in that manner. He knew that this would raise a storm of objections. However, he would ask the question, "Shall we take out the first bicuspid in this case?"

Dr. Bogue stated that all the light he was able to throw on Dr. Stockton's case would be against any extraction. The teeth being unilaterally dislocated, the easier and more simple method would be to place them in the position intended by nature that they should occupy. Any attempt to thwart nature would be met with indifferent success. Such an extraction would also diminish the nasal cavity, the oral cavity, and the size of the dental arches, diminishing the young man's dignity of countenance.

Dr. Bogue also presented a case of irregularity upon which he invited comment. The left lower bicuspids did not articulate with anything. The right lower second molar was also turned in such a way that it did not articulate, with anything. The upper molars were also both dislocated anteriorly. The teeth all stood apart so that nothing could be gained by extraction, and the upper teeth were so prognathous that the lips could scarcely be closed even with great effort.

Dr. S. E. Davenport wished to say a word to brother dentists who might, during the vacation season, be the means of helping the patients of others out of a difficulty. Dr. Davenport had his acknowledgments to make to a dear friend for services rendered to one of his pet patients during his absence. He wished to couple his acknowledgments with those of the patient for the kind and skilful way in which the service had been rendered. But Dr. Davenport had one fault to find. After the broken tooth had been made comfortable, the patient made her thanks and asked if she might be informed how much she was indebted for the service. The dentist declined to accept a fee, saying that he was glad to be of service to his friend Dr. Davenport. It was upon this point that Dr. Davenport wished to speak. He had always considered that this was a wrong method to pursue in such cases, and it had a tendency to belittle in the patient's mind the value of such service. He believed that a charge should be made as though the services were rendered by the patient's own dentist. So far as he personally was concerned, he should ask his friends so called upon during his absence to make a proper charge in such instances, or, at least, to inform him upon his return of the incident, so that the proper fee might be added to the patient's account.

The President thought the remarks of Dr. Davenport were very proper and timely. Many cases of this kind occur where patients are temporarily relieved by other dentists than their own, without fee, although they expect and desire to pay. He thought this a mistaken idea of conferring a favor upon a fellow-practitioner. No other professional men have such notions, and it seems explicable in the case of dentists only by remembering that as a profession we are young. The value of the service rendered is minimized in the patient's estimation by such practice, and neither practitioner is benefited.

Dr. J. Bond Littig did not think Dr. Davenport had taken just the right view of the matter. If Dr. Davenport's patient came to him for treatment he would say to him that he was not doing this for him, but for Dr. Davenport.

Dr. Wheeler thought it was customary among physicians to do the temporary service and then pass the account over to the physician who rendered the bill. However, he thought Dr. Davenport was right, more especially as by following the opposite course we were putting another man's patient under obligation to us, which we should be averse to do. Personally he would desire any patient of his who received treatment elsewhere to be charged a regular fee.

Dr. Littig said that this was not exactly the point. In rendering this bill the fee might be less than the patient had been in the habit of paying, which might cause dissatisfaction between the patient and his dentist. He thought it better to let the patient's dentist render a bill for the services.

Dr. R. T. Moffatt, of Boston, read a paper entitled "The Art of carving and baking Porcelain Teeth and Crowns."

(For Dr. Moffatt's paper, see page 1.)

DISCUSSION.

Dr. J. Bond Littig did not think he could add anything in the way of suggestions or criticisms, as the essayist had presented the subject in such a concise manner. The paper had carried him back forty years, when the materials for this work used to be ground up by him in a mortar, the quartz, the spar, and the kaolin. At that time very little kaolin was used on account of the opacity it produced. Kaolin was simply disintegrated felspar, having been deprived of its potash and soda, and so was infusible. One great objection to the carved teeth was that they were not as strong as the moulded teeth, because they were not made under such pressure. It used to be a very difficult piece of work to bring out whole one of these carved sections of six teeth after vulcanizing. It was the custom to make a full set of teeth in three sections, the bicuspids and molars in one section and the six front teeth in another. In carving the crown for the space mentioned by Dr. Moffatt, he did not state how much wider it was necessary to make the tooth to allow for contraction after baking.

Dr. C. S. Stockton was astonished to find that what he had considered as almost a lost art was still practised with such success.

However, the question arose, Could we make such a technical knowledge as that possessed by Dr. Moffatt of any value to us, practically, in our every-day practice? He himself had had a thorough training in this work, having carved thousands of sets of teeth, but, from a practical stand-point, to-day the knowledge was useless to him. There were occasions, however, when it was well to know how to carve teeth. He thought it might be made more available now that there had been such an improvement in furnaces, and, inasmuch as the dental course at college had been increased to four years, it would be well to take a little of this time in teaching young men how to carve teeth as the older ones here to-night had been taught years ago.

Dr. Moffatt, in answer to Dr. Littig's suggestion about not using much to make it opaque as to increase the plasticity as an aid in about twenty grains to the ounce of body. It was not put in so much to make it opaque as to increase the plasticity as an aid in carving. Dr. Moffatt thought possibly the carved teeth might not be quite as strong as teeth made under great pressure, but under practical tests in removing the pins from different moulded teeth he had found it more difficult to break carved teeth than "store" teeth. He did not think the question of burning out the color in baking was worth considering, as with a little practice this could always be regulated. Regarding the question of shrinkage in the crown mentioned, the shrinkage in this case took place longitudinally entirely. Dr. Moffatt did not think the age was too fast to appreciate this kind of work. He was sure that thorough work was worth the time given to it.

Dr. Wheeler thought that the proportion of fusible material in a body greatly affected its shrinkage while baking, and that colors were also retained better in the higher fusing bodies, as, for instance, the Parker body as compared with the S. S. White. It seemed to him that this question of carved *versus* moulded teeth was merely the question of work done by an artist compared with work done by an ordinary factory moulder. He thought some of the material furnished us by the supply houses was just about as near artistic perfection as we would expect from a foundry laborer getting two or three dollars a day. The colleges had been playing into the hands of the supply houses in not having such a course in the regular college work. He did not believe that carved teeth were weaker. In his experience they were even stronger. Teeth that were carved contained a larger percentage of body than the moulded teeth, and this would perhaps account for their greater strength, the enamel being much weaker, and in moulded teeth it occupied a much greater proportion. He thought that Boston, with its several practitioners engaged in this work of carving teeth, would compare very favorably with Philadelphia, the Mecca of supply houses, and where, to his knowledge, there was not a single man who could carve a set of teeth.

Dr. Littig thought it required special adaptability to do this work. He did not think, out of an ordinary class of one hundred, over twenty could be taught to do that kind of work. It was hard enough to teach them to make a plaster model. The college takes the trend of general practice throughout the country, and there were very few men now who did this work to any extent whatever.

The President thanked Dr. Moffatt for presenting the subject in such a careful manner and with such illustrations that any one may become well informed in this work.

Dr. Bogue stated that at one time Dr. Moffatt's father was his partner on the other side of the water. He was a man to whom we could all go to school and learn. Dr. Bogue recalled many instances in which Dr. Moffatt, Sr., had tried to teach him to do this beautiful work. He was exceedingly gratified to find that Dr. Moffatt had given the Institute such a paper from which the younger men could gain a knowledge of work that in after years would be their consolation and delight.

Dr. Davenport said he would like to make his thanks to Dr. Moffatt for doing what he had for the Institute. Dr. Moffatt was invited by the Executive Committee to favor the society with a paper, and the result had been most satisfactory. He had brought a great deal of paraphernalia from Boston which perhaps some would have never seen otherwise. Dr. Moffatt had given a clinic during the afternoon attended by at least twenty-five gentlemen, where he had done quite a little of this beautiful work. The members of the society were greatly indebted to Dr. Moffatt for the complete and earnest manner in which he had served them.

Dr. Strang would like advice concerning a very puzzling case he had at present under treatment. About a month ago a lady, thirty years of age, had been sent to him by a physician. The left side of the face was very much swollen and there was considerable discharge exteriorly from a fistula. There had been practically no motion of the inferior maxilla for three months, and she had been unable to take solid food. The history was that about one year ago she had had the lower second molar on that side filled, and subsequently the tooth gave so much trouble that it had to be extracted. The swelling began at that time. Dr. Strang had opened into the abscess at one point on the cheek, the pus having come almost to the surface, and had gotten out about a spoonful of what used to be called "laudable" pus. By inserting first a wood and then a rubber wedge he was able to get the mouth open sufficiently in two days to make an examination, and he was convinced that it was arsenical poisoning due to careless manipulation while devitalizing a pulp. There were discharging at the present time three fistulæ opening on the cheek, besides a discharge of pus at the posterior part of the sixth-year molar. He had scraped away a little of the bone that was soft and could be removed without pain to the patient. The temperature had been taken twice a day, and remained about normal. The patient is pregnant, and expects to be confined early in November.

The question is, Will it be judicious to extract the sixth-year molar?

The President thought the case probably called for the removal of the molar, which may be aggravating the morbid conditions. He supposed from the description that there must be considerable necrosed bone, but that it had not yet shown any demarcation. The removal of the tooth may be a needed preliminary. A sulphuric acid (about 1 in 15 or 20 of water, or the so-called aromatic sulphuric acid) treatment he had found effectual in hastening exfoliation. It was the custom of many surgeons to scrape or bur away the necrosed bone, but he thought it better to let nature's forces cause the separation, which they will do if helped.

Dr. Bogue suggested the cutting process for the removal of a greater portion of the necrosed bone.

Dr. Leo Green said that, inasmuch as three fistulæ were present, marking out a triagular area of which they were the apices, it would seem that the necrotic region had become circumscribed, and that the sequestrum was about to be exfoliated. It would be found floating in pus and could be easily removed.

Adjourned.

Fred. L. Bogue, M.D., D.D.S.,
*Editor The New York Institute of Stomatology.*

